# Acute frailty services: results of a national day of care survey

**DOI:** 10.1186/s12877-024-05075-1

**Published:** 2024-07-16

**Authors:** Vicky Kamwa, Thomas Knight, Catherine Atkin, Tim Cooksley, Chris Subbe, Mark Holland, Daniel Lasserson, Elizabeth Sapey

**Affiliations:** 1 Birmingham Acute Care Research Group, Institute of Inflammation and Ageing, NIHR Applied Research Collaborative West Midlands, NIHR Patient Safety Research Collaborative West Midlands, University of Birmingham, Birmingham, England; 2 The Christie NHS Foundation Trust, Manchester, England; 3 School of Medical and Health Sciences, Bangor University, Bangor, Wales; 4 School of Clinical and Biomedical Sciences, University of Bolton, Bolton, England; 5 Warwick Medical School, University of Warwick, Coventry, England

**Keywords:** Acute medical unit, Acute frailty services, Frailty, SAMBA, Prevalence

## Abstract

**Introduction:**

Older people living with frailty are at high risk of emergency hospital admission and often have complex care needs which may not be adequately met by conventional models of acute care. This has driven the introduction of adaptations to acute care pathways designed to improve outcomes in this patient group. The identification of differences in the organisational approach to frailty may highlight opportunities for quality improvement.

**Methods:**

The Society for Acute Medicine Benchmarking audit is a national service evaluation which uses a single day-of-care methodology to record patient and organisational level data. All acute hospitals in the United Kingdom are eligible to participate. Emergency admissions referred to acute medical services between 00:00 and 23:59 on Thursday 23rd June 2022 were recorded. Information on the structure and operational design of acute frailty services was collected. The use of a validated frailty assessment tool, clinical frailty scale within the first 24 h of admission, assessment by an acute frailty service and clinical outcomes were reported in patients aged 70 year and above. A mixed effect generalised linear model was used to determine factors associated same-day discharge without overnight stay in patients with frailty.

**Results:**

A total of 152 hospitals participated. There was significant heterogeneity in the operational design and staffing model of acute frailty services. The presence of an acute frailty unit was reported in 57 (42.2%) hospitals. The use of validated frailty assessment tools was reported in 117 (90.0%) hospitals, of which 107 (91.5%) used the clinical frailty scale. Patient-level data were recorded for 3604 patients aged 70 years and above. At the patient level, 1626 (45.1%) were assessed using a validated tool during the admission process. Assessment by acute frailty services was associated with an increased likelihood of same-day discharge (adjusted OR 1.55, 95%CI 1.03- 2.39).

**Conclusion:**

There is significant variation in the provision of acute frailty services. Frailty-related policies and services are common at the organisational level but implemented inconsistently at the patient level. Older people with frailty or geriatric syndromes assessed by acute frailty services were more likely to be discharged without the need for overnight bed-based admission.

**Supplementary Information:**

The online version contains supplementary material available at 10.1186/s12877-024-05075-1.

## Introduction

Frailty is conceptualised as a state of vulnerability to endogenous and exogenous stressors caused by changes in homeostasis related to ageing [1]. Frailty is associated with increased risk of adverse outcomes following hospital admission, including mortality, increased length of stay and readmission [2–5]. Frailty is also associated with hospital acquired complications such as deconditioning, delirium, falls and pressure ulcers [3, 6, 7]. Estimates of the prevalence of frailty in contemporary studies of hospitalised adults vary between 30 and 60% based on the method of measurement and population included [2, 8, 9].

A variety of tools and methods of assessment have been developed to identify frailty in the acute care setting [10, 11]. The Clinical Frailty Scale (CFS) is a validated frailty assessment tool which can be used to identify frailty using functional impairment as a surrogate marker [12]. The CFS was originally validated in the community setting but has since been widely applied in the acute hospital setting [13]. The use of CFS during the admission process may allow the early identification of patients likely to benefit from interventions, such as comprehensive geriatric assessment (CGA) or continued inpatient care in specialist units [14, 15]. CGA is a multidimensional assessment which highlights medical, social and functional needs and leads to the formulation of a patient-centred integrated care plan [16].

The National Health Service (NHS) has recognised the potential benefits of a coordinated organisational response to frailty and has issued policy documents mandating the development of acute frailty services. The NHS Long Term Plan states that all hospitals with a Type 1 Emergency Department (ED) should provide an acute frailty service for at least 70 h a week [17]. Acute frailty services should be composed of a multi-disciplinary team capable of providing all domains of CGA. NHS England and NHS improvement (NHSE/I) have published additional guidance to support the implementation of acute frailty services alongside a series of metrics to measure performance [18]. The guidance states all patients over the age of 65 should be assessed using the CFS within 30 min of arrival to hospital. Recognition of severe frailty (CFS 7 to 9) should trigger assessment to diagnose the presence of co-existent geriatric syndromes within one hour and prompt consideration of end-of-life care. The guidance advocates early access to CGA as means to reduce the need for hospital admission and facilitate same-day discharge [18]. The recommendations do not specify how services should be configured locally. There is no consensus on the optimal configuration of acute frailty services. A systematic review of acute frailty services highlighted significant variation in the populations targeted, the interventions delivered and reported outcomes [19]. The relevant literature is dominated by single centre observational studies at high risk of bias.

We aimed to describe variation in the operational approach to acute frailty across hospitals in the United Kingdom (UK). We quantified the prevalence of frailty assessment using validated frailty assessments tools at the organisational and patient level. Factors associated with same-day discharge were identified amongst a population of patients with frailty. This was achieved using the Society for Acute Medicine Benchmarking Audit (SAMBA), a national audit of acute medical care using a single day-of-care methodology [20, 21].

## Methods

### Ethics approval and consent to participate.

SAMBA is registered as a priority audit on the NHS England Quality Accounts list. All data is collected in the delivery of routine care. The North-West Wales Ethics Committee confirmed that the SAMBA did not require formal ethical review and formal consent from participants was not required. Participating sites register with the Society for Acute Medicine and follow local audit registration approval processes. Local Caldicot guardian approval is required to participate. No identifiable data is transferred from participating sites. Health Research Authority has been granted to allow secondary analysis on non-identifiable data (REC 21/HRA/4196).

### SAMBA methodology

SAMBA is a cross-sectional service evaluation conducted by the Society for Acute Medicine annually on the penultimate Thursday in June. All hospitals in the UK receiving acutely unwell (non-elective, adult) medical patients are eligible to participate. Non-acute and community hospitals are excluded. The SAMBA22 protocol and data collection forms are publicly available [22]. Study reporting followed the Strengthening reporting of observational studies in epidemiology (STROBE) guidelines [23].

SAMBA consists of two separate components completed by each participating hospital. An organisational survey was distributed to the designated SAMBA lead in each participating hospital one week prior to collection of patient level data. The survey contains questions relating to the organisational structure of acute frailty services, the range of services provided and their staffing model. The survey was completed by a locally designated SAMBA lead.

Patient level data were collected on Thursday 23rd June 2022 between 00:00 and 23:59. All patients referred to acute medical services during the study period were eligible for inclusion. Data were collected electronically using a web-based data collection interface [24]. Patient level data were collected by named members of the acute medicine team. All variables were collected during the delivery of routine care and ascertained by retrospective review of the clinical record.

The patient level evaluation contained multiple variables relevant to acute care for older people living with frailty. These included whether assessment using a validated frailty assessment tool had been recorded during the admission process and the outcome of the assessment (recorded as a dichotomous, frail vs non-frail based on the local threshold in operation). If CFS was not recorded during the admission process, it was determined by the data-collector using available information from the clinical records. Retrospective determination of CFS using case notes review has previously shown to have reasonable agreement with prospective determined values [25]. This provided a quantitative measure of frailty less dependent on the operationalisation of frailty screening at the hospital level. The data collection tool provided data collectors with a visual guide to calculating CFS and explicitly stated the CFS should be calculated based on functional performance 2 weeks prior to admissions. SAMBA also recorded whether the patient had a geriatric syndrome (falls, cognitive impairment, delirium, new urinary incontinence) as a primary or contributory cause of admission [26]. Patient outcomes were recorded by review of the case notes or electronic health record at 14 days after admission.

Organisational survey data and patient level data was used to describe variation in acute frailty service provision. The operational characteristics of acute frailty services, including eligibility criteria and staffing models were described. Patient level analysis relating to the prevalence of frailty assessment during the admission process was restricted to people aged ≥ 70. National guidelines recommend routine assessment for frailty in patients aged ≥ 65 [18]. SAMBA records age in 10-year bands which precluded assessment in this specific age range.

A subgroup of patients with feature of frailty was defined to determine the relationship between acute frailty service review and same-day discharge. Criteria for the sub-group with features of frailty included CFS ≥ 5, the presence of a geriatric syndrome or both. In circumstances where the CFS and geriatric syndrome were unrecorded, receipt of a package of social care or residence in nursing or residential care was used as a proxy for frailty. Patients with a CFS < 5 and without a geriatric syndrome, or where both variables were unrecorded, not in receipt of a social package of care or residing in nursing or residential care were considered not to have frailty and were excluded from the sub-group analysis.

### Maps

Geographical variation in the provision of acute frailty services was visualised using a series of maps. Boundary data was obtained from the Office of National Statistics geoportal to facilitate comparison between regions (Integrated Care Systems in England, local Health Boards in Scotland and Wales and Health and Social Care Trusts in Northern Ireland. SAMBA participation rate was calculated using the number of hospitals with a Type 1 Emergency Department in each jurisdiction as the denominator. Maps were created in R studio using R statistical software (Version 4.1.2., Vienna. Austria). Geospatial files were manipulated using the geojsonio package.

### Statistical analysis

Continuous data are described using the mean and standard deviation for normally distributed data and the median and interquartile range (IQR) for non-normally distributed data. Outcome variables were described using counts and proportions. Differences between proportions were compared using a chi-square test. Spearman rank order tests were used to assess correlation between variables.

### Mixed effect logistic regression models

A multi-variable mixed effect logistic regression models was specified to assess factors association with same-day discharge amongst the sub-group with frailty. Hospitals were incorporated as a random intercept to represent the hierarchical structure of the data. Age (in 10 years bands), arrival to hospital out of hours (between 20:00–07:59), initial assessment in a same-day emergency care area, the presence of a geriatric syndrome and the National Early Warning Score 2 (NEWS2) were used as predictor variables in the model. NEWS2 values were dichotomised based on NEWS2 ≥ 4, which is used in national guidelines to prompt urgent assessment and consideration of escalation of clinical care and enhanced monitoring [27]. A NEWS2 ≤ 4 has been proposed as threshold at which same-day discharge may be considered appropriate in selected patients [27, 28].

The random intercept was used to obtain statistically valid co-efficient given the hierarchical clustered data structure but was also used to quantify variation at the hospital level [29]. To provide a more intuitive representation of variation between hospitals the random effects on the log-odd scale were converted to a median odds ratio (MOR) [30]. The MOR is the median value of the odds ratio (OR) of all possible pair-wise comparisons with the highest OR as the numerator in all pairs. Confidence intervals were created for the MOR using a parametric bootstrap technique using 1000 replicates. Fixed effect coefficients are reported as odds ratios (OR) with 95% confidence intervals calculated using a Wald test. The reported fixed effects are conditional on the random effect and represent the effect of the variable comparing patients from the same hospital rather than the average (marginal) effect. Generalised mixed effect models were implemented in the lme4 package using R statistical software (Version 4.1.2., Vienna. Austria).

## Results

### Description of data

A total of 152 hospitals contributed to SAMBA22. The participation rate was 63.4% across all eligible UK hospitals. Participation rate varied across the UK, England 76.7% (*n* = 135), Scotland 27.6% (*n* = 8), Wales 46.1% (*n* = 6), and Northern Ireland 25.0% (*n* = 3). Organisational data in relation to the provision of frailty services was provided by 128 (84.2%) hospitals. Patient level data without accompanying organisational data was provided by 9 (6.0%) hospitals. Partial completion of the survey data missing responses to survey questions specifically relating to acute frailty services were provided by 15 (10.3%) hospitals. The median total hospital inpatient bed number in participating hospitals was 520 (IQR 387–680) and the median number of AMU beds was 40 (IQR 30–52). The mean number of admissions per participating hospital was 48 (SD 22.1) and the mean number of admissions of people aged ≥ 70 was 29 (SD 12). Patient level data was collected for 7248 patients of which 3604 (49.7%) were aged ≥ 70 years. The proportion of patients aged ≥ 70 of the total recorded ranged from 7.7% to 97.1% at the hospital level.

### Use of validated frailty assessment tools

#### Hospital level

A policy to assess for frailty using a validated tool during the admissions process was present in 117 (90%) hospitals. This included the provision of training on the use of the tool in 62 (52.9%) hospitals. CFS was used to assess for frailty in 107 (91.5%) hospitals. The outcome of frailty assessment was used to stream patients to different clinical care pathways in 56 (47.9%) hospitals. Assessment was documented in the clinical notes during medical assessment in 64 (54.7%), during initial triage in 23 (19.7%) hospitals, as part of a nursing assessment bundle in 18 (15.3%). The process of assessment was not described in 12 (10.3%) hospitals. The outcome of frailty assessment was recorded electronically in 71 (60.7%) hospitals.

#### Patient level

Frailty assessment using a validated tool during the admission process was recorded in 1626 (45.1%) patients aged > 70. The proportion of patients assessed using a validated tool ranged from 0 to 100% within individual hospitals. Frailty was identified in 1141 (70.2%) assessed using a validated tool (as defined using local operational criteria). The outcome of assessment was not reported in 27 (1.6%). No frailty assessments were recorded at the patient level in 15 (12.8%) hospitals that reported the use of a validated frailty assessment tool in the organisational survey. Frailty assessment was recorded in 1368 (48.0%) patients initially assessed in the ED and 106 (24.3%) initially assessed in SDEC. There was no statistical difference in the proportion of patients assessed for frailty during the admission process comparing hospitals that reported using the tool to stream patients to appropriate downstream care pathways with those that did not (used to stream, 48.1%, did not use to stream 52.1%, p value 0.06).

CFS was recorded retrospectively in 2730 (75.7%) patients aged ≥ 70 years. The CFS variable was not recorded in 874 (24.3%) patients. The CFS was ≥ 5 in 1319 (48.3%) patients. In the patient group not assessed using a validated tool during the admission process the retrospectively determined CFS was ≥ 5 in 490 (37.1%) patients. A geriatric syndrome was recorded as being present in 1346 (37.3%) patients aged ≥ 70 years. The geriatric syndrome variable was not recorded in 98 (2.7%) patients. In the patient group not assessed using a validated tool during the admission process, a geriatric syndrome was present in 586 (30.8%) patients.

### Acute frailty services provision

A range of services and capabilities were identified with a focus on older people living with frailty. The provision of no acute frailty or community services as defined in the organisational survey was reported in 14 (10.6%) hospitals. Multiple services were often delivered within the same hospital. Elements of CGA could be completed in the community using a discharge to assess model in 75 (56.8%). The option to transfer patients to an intermediate care facility directly from the ED was available in 74 (56.5%) of hospitals. Ring-fenced nursing home beds were reported in 21 (15.9%) hospitals. The frequency of individual services and their combinations are shown in Fig. 2A. Hospitals frequently had access to multiple frailty services located in both hospital and community settings with no clearly discernible pattern in the combination of services provided.

### Comprehensive Geriatric Assessment (CGA) in the Emergency Department (ED)

**Fig. 1:**
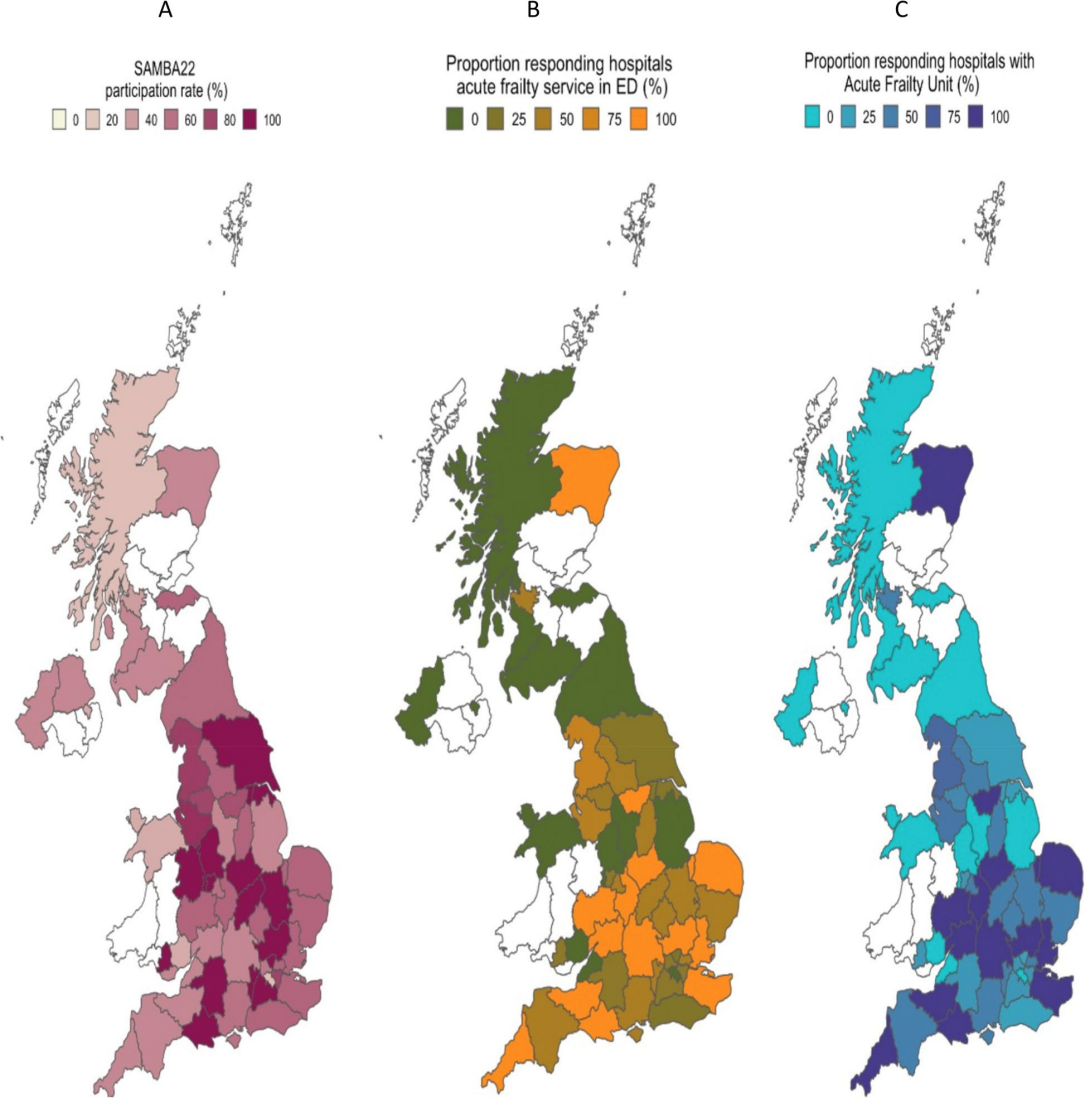
Geographical variation in SAMBA22 participation and acute frailty service provision in the UK. A: SAMBA22 participation rate. B: Proportion of hospitals providing frailty in-reach services to ED. C: Proportion of hospitals with an acute frailty unit. **A** shows the proportion of hospitals within each geographical region that participated in SAMBA22. **B** shows the proportion of SAMBA participating hospitals within each geographical region that reported the presence of an acute frailty ED in-reach service. **C** shows the proportion of hospitals within each geographical that reported the presence of an acute frailty unit. Geographical areas with no SAMBA participating hospitals or absent fields to organisation survey questions relating to frailty series are coloured white across all maps. Geographical boundaries relate to local NHS organisational structure. NHS England Integrated. Care Systems (*n* = 42). NHS Scotland Health Boards (*n* = 14), NHS Wales local health boards (*n* = 7). Geographical variation in the prevalence ED frailty in-reach services and acute frailty should be interpreted in the context of variation in participation rate illustrated in Fig. 1A

The provision of CGA in the ED by an acute frailty multi-disciplinary team was reported in 80 (61.3%) hospitals. The geographical distribution of CGA provision in ED is provided in Fig. 1A. The acute frailty service also provided in-reach into the AMU in 45 (56.3%) hospitals and into the Ambulatory Emergency Care area in 39 (48.8%) hospitals. The operational hours of the service varied, 35 (43.8%) operated between core hours (08:00 – 17:00) and 41 (51.3%) also operated outside core hours (operational after 17:00), of which 2 (2.5%) reported a 24-h service. Operational hours were not provided by 5 (6.3%) hospitals that provided CGA in ED.

**Fig. 2:**
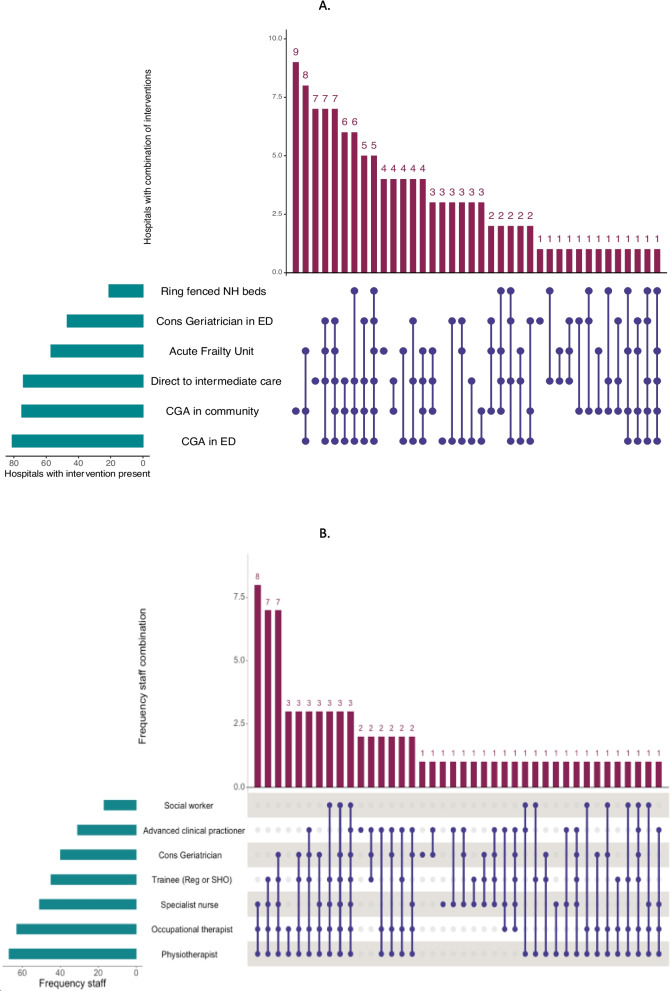
Upset plot showing the frequency of service provision and staffing mix of acute frailty services. **A** Adaptations to acute care pathways related to frailty; **B** Staffing models of acute frailty services in the Emergency Department. UpSet plots allow visualisation of data with intersecting sets. Horizontal bars in green represent the total number of hospitals reporting the service or staff member. The central portion of the plot contains purple lines and dots, representing specific combinations of services or staffing models. The violet vertical bars at the top of the plot represent the total number of hospitals with the specific combination of interventions or staffing model represented by the purple line and dots directly below. For example, in Fig. 2B. The bottom green horizontal bar illustrates 67 hospitals include a physiotherapist, the most leftward vertical violet bar illustrates there are 8 hospitals that are staffed by a specialist nurse, occupational therapist and physiotherapist as indicated by the purple line and dots immediately below

A range of acute frailty service staffing models were identified and are summarised in Fig. 2B. A consultant geriatrician was present within the ED in 47 (36.7%) hospitals. Physiotherapists were the most represented professional group within services providing CGA in the ED and were present in 67 (52.3%) hospitals. Social workers were the least represented professional group, present in 17 (13.2%) hospitals. Three broad staffing models could be differentiated, teams comprised exclusively of nurses and allied health care professional without doctors as part of the immediate team (*n* = 17, 21.3%), teams comprised exclusively of doctors and advanced care practitioners (*n* = 6, 7.5%) and teams with a mixture of medical, nursing and allied health professionals (*n* = 57, 71.3%).

Different approaches to referral and patient selection were evident in the organisational survey data. The option to stream older people directly to an acute frailty MDT without prior medical assessment in the ED was present in 36 (44.4%) services. Patients were screened directly by members of the acute frailty team in 24 (29.6%) services, referred after assessment in ED in 26 (32.1%) service, referred following assessment by the medical team in 11 (13.5%) services and direct from ED triage in 8 (9.8%) services. A combination of the above processes was reported in 10 (12.3%) services.

CFS was utilised within criteria to select patients for acute frailty service review in 61 (76.3%) hospitals.

The most common CFS threshold was ≥ 5 (CFS ≥ 4, *n* = 14 (22.9%), CFS ≥ 5, *n* = 24 (39.3%), CFS ≥ 6, *n* = 8 (13.1%)).

The CFS threshold was not reported in 15 (24.5%) hospitals. Age criteria were utilised in 51 (63.8%). The most common age threshold was ≥ 75 (≥ 60, *n* = 1 (2.0%),. ≥ 65, *n* = 14 (27.5%), ≥ 70, *n* = 1 (2.0%), ≥ 75, *n* = 25 (49.0%), ≥ 80, *n* = 5 (9.8%)) The age threshold was not reported in 5 (9.8%) hospitals.

### Acute frailty units

Acute Frailty Units (AFU) were reported in 57 (42.2%) hospitals. The geographical distribution of AFU is demonstrated in Fig. 1B. AFUs had a range of operational structures, 45 (78.9%) were independent wards, geographically separated from the ED and AMU, 6 (10.5%) were allocated beds located within the AMU, 1 (1.8%%) unit used allocated beds within a short stay area of the ED and 4 (7.0%) employed a SDEC model with no capacity for overnight stay. Environmental adaptations, such as non-slip flooring had been made in 26 (45.6%) AFUs.

The most common route of entry to the AFU, reported in 34 (59.6%) hospitals was following assessment by the ED or medical team. AFU accepted referrals directly from primary care in 26 (45.6%) hospitals and directly from the ambulance service in 14 (24.5%) hospitals. CFS was used to identify eligible patients in 37 (54.4%) acute frailty units. The most commonly utilised CFS threshold was ≥ 5 (CFS ≥ 4, *n* = 8 (21.6%), CFS ≥ 5, *n* = 12 (32.4%), CFS ≥ 6, *n* = 6 (16.2%)). The CFS threshold was not reported in 11 (29.7%) AFUs.

### Assessment by acute frailty services

**Fig. 3:**
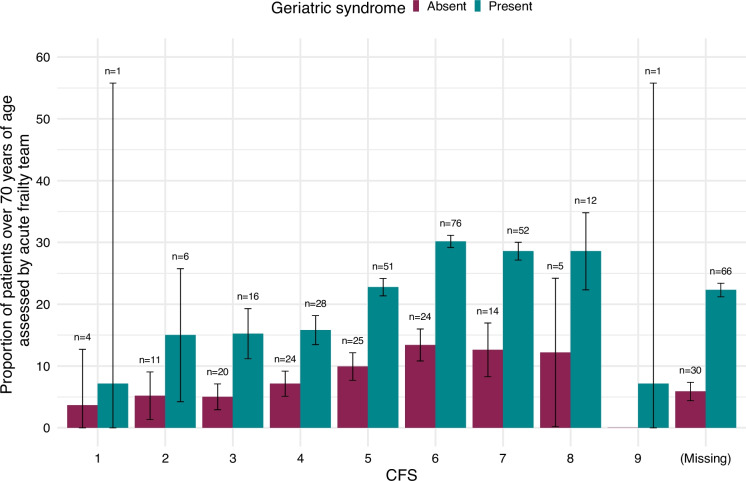
Proportion of patients aged > 70 assessed by acute frailty services stratified by CFS and the presence of frailty. The number of patients within each bar is reported above the error bar. Patients with missing data in relation to the CFS were retained in the plot. Error bars represent 95% confidence intervals. CFS: clinical frailty scale.

Assessment by an acute frailty service was recorded in 471 (13.0%) of patients aged ≥ 70. Acute frailty service review was undertaken in 321 (28.6%) of patients identified as frail using a validated tool during the admission process. Acute frailty service review was recorded in 309 (23.4%) of patients with a geriatric syndrome and in 263 (20.0%) of patients with a recorded CFS ≥ 5. The geriatric syndrome variable was not recorded in 119 (3.0%) patients. The prevalence of assessment increased incrementally with increasing CFS and plateaued at scores ≥ 6. (Fig. 3). In hospitals that recorded at least one assessment by an acute frailty service the median number of assessments was 3 (IQR 1—7). There was a weak positive correlation between the proportion of patients aged ≥ 70 assessed using a validated assessment tool and the proportion assessed by acute frailty services (spearman coefficient 0.26, *p* value < 0.05) in hospitals that recorded at least one assessment by an acute frailty service.

### Factors associated with acute frailty team review and same-day discharge

Sub-group analysis was undertaken on the patient population with features suggestive of frailty. A total of 2142 59.4% of aged ≥ 70) people met the criteria for frailty. A summary of the characteristics of the sub-group is provided in supplemental Table 1. No features suggestive of frailty (absence of a geriatric syndrome, CFS ≥ 5 and not in receipt of social package of care) were recorded in 1462 (40.5%). Discharge without overnight stay was achieved in 204 (9.5%) patients.

**Fig. 4:**
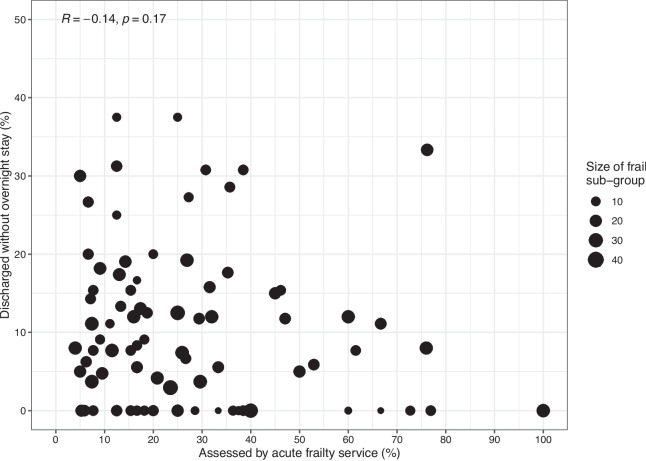
Association between the proportion of patients assessed by acute frailty services and the proportion of patients discharged without overnight admission at the hospital level amongst the sub-group of patients with frailty. The size of dots in the scatter plot are proportional to the size of the sub-group with frailty within each individual hospital.

At the hospital level there was no statistically significant correlation between the proportion of patients assessed by acute frailty service and the proportion of patients discharge without overnight stay (correlation -0.014, p value 0.17) (Fig. 4). Assessment by acute frailty services was associated with an increased likelihood of same day discharge (adjusted OR 1.55, 95%CI 1.03- 2.39). There was statistical evidence of variation in the likelihood of same-day discharge between hospitals (hospital level random effect: MOR 1.70, 95% CI 1.83–1.97). Output from the regression model is provided in Table 1

**Table 1:** Regression coefficients

Variable	Odds ratio	95% confidence interval	*P* value
Intercept	0.14		
Assessed by acute frailty team	1.55	1.03 – 2.39	< 0.05
Age	0.96	0.75—1.29	0.75
NEWS2 ≤ 4	586 (82%)	0.13—0.44	< 0.001
Presence of geriatric syndrome	0.45	0.32—0.64	< 0.001
Arrival out of hours (20:00:07::59)	1.44	0.99–2.09	0.06
Initial assessment in SDEC	19.6	11.9—32.4	< 0.001
Random effect: variance	3..29		
Random effect: Median OR	1.70	1.83–1.97	

Output from the generalised linear model. Regression coefficients are expressed as odds ratios. Fixed effect coefficients are reported as odds ratios (OR) with 95% confidence intervals calculated using a Wald test. The reported fixed effects are conditional on the random effect and represent the effect of the variable comparing patients from the same hospital rather than the average (marginal) effect. OR: odds ratio. SDEC: same day emergency care, NEWS2: National Early Warning Score 2

## Discussion

This nationwide cross-sectional day of care of evaluation provides evidence of significant variation in the provision of acute services for older people living with frailty at the system level within the United Kingdom (UK). Our findings suggest a gap between the aspirations of national policy and the reality of clinical practice. The provision of acute frailty services is not universal and there are notable differences in the operational design and implementation of services at the hospital level. There also appears to be discordance between work as imagined, reflected in the organisational survey responses and the actuality of clinical practice. These observations may help guide quality improvement efforts and inform debate on the optimal configuration of acute frailty services. AFUs differ from traditional bed-based care by incorporating the principles of same-day emergency care to deliver earlier access to CGA and diagnostic services to reduce length of stay. Less than half of hospitals had access to a dedicated AFU.

Assessment by acute frailty services was associated with an increased likelihood of same-day discharge amongst patients with frailty. The direction of causality cannot be inferred from our results. The possibility that acute frailty services were instrumental in achieving same-day discharge and the possibility that acute frailty services preferentially assess older people more likely to go home are both compatible with our observations.

A recent randomised controlled trial of CGA delivered in the ED demonstrated a significant reduction in ED length of stay and reduced the need for hospital admission suggesting early intervention at the front door has a direct impact on outcome [31]. A narrow focus on same-day discharge as a metric of efficacy is not without risk.

Services that target lower acuity patients in the pursuit of same-day discharge may inadvertently divert attention and resources from more complex patients with severe acute illness who stand to gain equally from multi-dimensional assessment and early rehabilitation.

Our study highlights only a relatively small proportion of older people with frailty or presenting with geriatric syndromes are assessed by specialist acute frailty services. There is a mismatch between the number of older people living with frailty admitted with acute medical illness who may be expected to benefit from CGA and the provision of specialised acute frailty services. The ability to provide elements of CGA is not confined to clinicians working in acute frailty services and these observations highlight the need for non-specialists to incorporate elements of CGA into routine practice. Older people living with frailty admitted to hospital with acute medical illness experience delays in clinical assessment [32] and longer waiting times in the ED [33]. Studies that explore the indirect impact of acute frailty services on the care of older who do not meet the criteria for acute frailty service assessment and receive routine care by the acute and general medical team are required.

Variation in the staff mix of acute frailty services and access to additional services suggest the organisational response to frailty may differ substantially between hospitals despite superficial similarities. There was significant variation in the likelihood of same-day discharge amongst patients with frailty between hospitals. Same-day discharge amongst older people with frailty was almost twice as likely on average (MOR 1.7) in pair-wise hospital comparisons. Whether differences in operational approach explain the observed variation is unclear. Older people living with frailty admitted to hospital for less than 72 h have comparable rates of emergency bed day use and survival to frail people admitted for longer periods [34]. Emergency admission of any length is therefore an adverse prognostic indicator in this group. It is vital that acute frailty services facilitating same-day discharge have access to enhanced community support, high quality rehabilitation services and the ability to undertake advance care planning in anticipation of future health crises [35, 36].

The number of hospitals incorporating frailty assessment tools into the admissions process at the policy level has increased from 62% in 2019 to 90% in the current study [37]. This has not been accompanied by an increase in the proportion of patients assessed at the patient level. Less than half of patients deemed at risk of frailty by age criteria are assessed at the patient level. This is consistent with previous estimates of the prevalence of frailty assessment on admissions suggesting the presence of unaddressed barriers to implementation [37]. Given we used an age threshold of 70 to define the population at risk this is likely to represent an overestimate of performance relative to national guidelines which recommend assessment for the presence of frailty in all patients 65 years old or above.

In the patient group not assessed using a validated tool during the admission more than 1 in 3 had a CFS ≥ 5 and 1 in 3 had a geriatric syndrome. This suggests an opportunity to systematically identify and quantify the severity of frailty at earlier time points in the acute care pathway is frequently missed. There is a robust evidence base which shows frailty assessment tools are good predictors of adverse outcomes including mortality, length of stay and the need for institutional care during and following emergency admission to hospital [4]. It is less clear how best to utilise this information to optimise the acute care process [13, 38]. Frailty assessment tools are not inherently useful and increasing the documentation of frailty assessment on admission should not be pursued as an objective for its own sake. Knowledge of the frailty status of a patient within the first 24 h of admission may provide an additional prompt to offer evidence-based interventions such as CGA [15, 39]. More consistent use of CFS may facilitate improved communication of the severity of underlying frailty in a simple and understandable way across care interfaces. The correlation between the proportion of older people assessed using a validated assessment tool on admission and assessment by acute frailty services within each hospital was weak. This suggests improved implementation of frailty screening does not directly translate to earlier specialist input. CFS is commonly used to select patients who may benefit from acute frailty service input, but CFS used in isolation identifies a population which greatly surpasses the current capacity of acute frailty service provision. This prompts questions as to whether the CFS is optimised to select patients who may benefit from acute frailty services on admission and whether more nuanced approaches to patient selection are used in practise.

### Strengths and limitations

The hospital participation rate in SAMBA22 allows a reasonable assessment of acute care delivery at the national scale. Around three quarters of hospitals in England contributed data, participation was lower in Scotland, Wales and Northern Ireland. The cross-sectional single day of care methodology employed in SAMBA has significant limitations. Case-mix adjustment was limited to crude measures of severity of illness and did not include detailed information in relation to diagnosis. Performance measured over a single day is likely to vary substantially at the individual hospital level due to random factors unrelated to the care model in operation. This risk of overstating variation was mitigated by aggregating hospitals within a mixed effect model. The magnitude of variation would be expected to decrease in a larger sample collected longitudinally. Acute frailty service assessment was associated with a statistically significant increase in the likelihood of same-day discharge, although there were wide confidence intervals around the estimate.

SAMBA is conducted as a service evaluation. This allows rapid data collection at scale at the expense of an increase in the risk of measurement error in comparison with a formalised observational study. Organisational survey responses were provided by a single individual at each participating hospital. There may have been systemic differences in how key variables such as CFS and the presence of geriatric syndromes were recorded between participating hospital. Organisational and patient level data contained missing variables. The CFS variable was omitted relatively frequently which precluded its use to define a population with frailty in isolation. Where relevant, outcomes are reported in the patient group with missing CFS values separately. There may be systemic differences between SAMBA participating and non-participating hospitals which may limit generalisation.

## Conclusion

There is significant variation in the provision of acute frailty services. Frailty related policies and services are common at the organisational level but implemented inconsistently at the patient level. Older people with frailty or geriatric syndromes assessed by Acute frailty services were more likely to be discharged without the need for overnight bed-based admission. It is unclear whether this reflects preferential selection of patients at higher likelihood of discharge or a direct effect of the intervention. Further research is required to evaluate the impact of frailty assessment and acute frailty services to help define features which drive improved clinical outcomes.

### Supplementary Information

Supplementary Material 1.

## Data Availability

The data that support the findings of this study are available upon reasonable request to support projects with the potential for patient benefit with permission of the Society for Acute Medicine. Data requests should be submitted to PIONEER@uhb.nhs.uk.
