# Supplementary material for: Acute frailty services: results of a national day of care survey
Source: BMC Geriatr. 2024 Jul 16;24:608. doi: 10.1186/s12877-024-05075-1 (PMC11251303; doi:10.1186/s12877-024-05075-1)
Supplement: Supplementary file 1 — Supplementary Material 1. [file 12877_2024_5075_MOESM1_ESM.docx]

Supplemental table 1:
Comparison of clinical characteristics of frail subgroup using study criteria.

| **Characteristic** | **CFS ≥ 5 & Geriatric syndrome present**  n = 714 | **CFS ≥ 5 & Geriatric syndrome absent**  n = 605 | **CFS <5 & Geriatric syndrome present**  n = 632 | **Receipt of social package of care**  n = 191 | **No features suggestive of frailty**  n = 1,462 | **p value** |
| --- | --- | --- | --- | --- | --- | --- |
| Sex (Female) | 313  (44%) | 270  (45%) | 297  (45%) | 88  (46%) | 739  (51%) | 012 |
| Age |  |  |  |  |  | <0.001 |
| 70-79 | 170  (24%) | 203  (34%) | 232  (38%) | 68  (3%) | 861  (59%) |  |
| 80-89 | 379  (53%) | 292 (48%) | 306 (48%) | 88 (46%) | 519 (35%) |  |
| ≥90 | 165 (23%) | 110  (18%) | 94 ( 15%) | 35  (18%) | 82  (6%) |  |
| NEWS2 ≤ 4 | 586  (82%) | 464  (77%) | 539  (85%) | 152 (80%) | 1,275  (87%) | <0.001 |
| Do Not Resuscitate order prior to admission | 257  (36%) | 182  (30%) | 118  (18%) | 54  (28%) | 86 (6%) | < 0.001 |
| Initial assessment in SDEC | 10  (1%) | 55  (9%) | 25  (4%) | 15  (8%) | 331  (23%) | < 0.001 |
| Assessed by acute frailty team | 192  (27%) | 71  (12%) | 117  (18%) | 25  (13%) | 66 (5%) | < 0.001 |
| Unknown | 5 | 13 | 19 | 13 | 69 |  |
| Outcome  (day 14) |  |  |  |  |  |  |
| Discharged without overnight stay | 36  (5%) | 82  (14%) | 52  (9%) | 34  (18%) | 449  (31%) | < 0.001 |
| Discharged before 14 days | 367  (51%) | 333  (55%) | 371  (59%) | 116  (60%) | 765  (52%) | < 0.001 |
| In-hospital (ICU admission) | 9 (1%) | 4  (1%) | 4  (1%) | 2 (1%) | 9 (1%) | 0.54 |
| In hospital at day 14 | 203  (28%) | 113  (19%) | 140  (23%) | 21  (11%) | 135 (9%) | < 0.001 |
| Readmitted | 7 (1%) | 7  (1%) | 6 (1%) | 2  (2%) | 12  (1%) | 0.9 |
| Self-discharge | 2 (<1%) | 3 (<1%) | 3  (< 1%) | 0 | 6  (<1%) | 0.86 |
| Died in hospital | 59  (8%) | 45  (7%) | 32  (5%) | 8  (4%) | 49 (3%) | <0.001 |
| Transferred to another health care facility | 26  (4%) | 15  (3%) | 23  (4%) | 5  (3%) | 27 (2%) | 0.07 |

*Legend: CFS = Clinical Frailty Score; POC = Package of Care; NEWS2 = National Early Warning Score 2; DNAR = Do Not Attempt Resuscitation; ED = Emergency Department; AMU = Acute Medical Unit; SDEC/AEC = Same Day Emergency Care/Ambulatory Emergency Care; SAMBA = Society for Acute Medicine Benchmarking Audit; ICU = Intensive Care Unit.*
